# Interlaboratory Evaluation of *in Vitro* Cytotoxicity and Inflammatory Responses to Engineered Nanomaterials: The NIEHS Nano GO Consortium

**DOI:** 10.1289/ehp.1306561

**Published:** 2013-05-06

**Authors:** Tian Xia, Raymond F. Hamilton, James C. Bonner, Edward D. Crandall, Alison Elder, Farnoosh Fazlollahi, Teri A. Girtsman, Kwang Kim, Somenath Mitra, Susana A. Ntim, Galya Orr, Mani Tagmount, Alexia J. Taylor, Donatello Telesca, Ana Tolic, Christopher D. Vulpe, Andrea J. Walker, Xiang Wang, Frank A. Witzmann, Nianqiang Wu, Yumei Xie, Jeffery I. Zink, Andre Nel, Andrij Holian

**Affiliations:** 1 Department of Medicine, Division of NanoMedicine, Center for Environmental Implications of Nanotechnology, California Nanosystems Institute, University of California at Los Angeles, Los Angeles, California, USA; 2 Center for Environmental Health Sciences, Department of Biomedical and Pharmaceutical Sciences, University of Montana, Missoula, Montana, USA; 3 Department of Environmental and Molecular Toxicology, North Carolina State University, Raleigh, North Carolina, USA; 4 Department of Medicine, University of Southern California, Los Angeles, California, USA; 5 Department of Environmental Medicine, University of Rochester, Rochester, New York, USA; 6 Department of Chemistry and Environmental Science, New Jersey Institute of Technology, Newark, New Jersey, USA; 7 Environmental Molecular Sciences Laboratory, Pacific Northwest National Laboratory, Richland, Washington, USA; 8 Department of Nutritional Science and Toxicology, University of California, Berkeley, Berkeley, California, USA; 9 Department of Cellular & Integrative Physiology, Indiana University School of Medicine, Indianapolis, Indiana, USA; 10 Department of Mechanical and Aerospace Engineering, West Virginia University, Morgantown, West Virginia, USA

**Keywords:** cell viability, inflammation, *in vitro*, MWCNT, nanotoxicology, round-robin testing, TiO_2_, ZnO

## Abstract

Background: Differences in interlaboratory research protocols contribute to the conflicting data in the literature regarding engineered nanomaterial (ENM) bioactivity.

Objectives: Grantees of a National Institute of Health Sciences (NIEHS)-funded consortium program performed two phases of *in vitro* testing with selected ENMs in an effort to identify and minimize sources of variability.

Methods: Consortium program participants (CPPs) conducted ENM bioactivity evaluations on zinc oxide (ZnO), three forms of titanium dioxide (TiO_2_), and three forms of multiwalled carbon nanotubes (MWCNTs). In addition, CPPs performed bioassays using three mammalian cell lines (BEAS-2B, RLE-6TN, and THP-1) selected in order to cover two different species (rat and human), two different lung epithelial cells (alveolar type II and bronchial epithelial cells), and two different cell types (epithelial cells and macrophages). CPPs also measured cytotoxicity in all cell types while measuring inflammasome activation [interleukin-1β (IL-1β) release] using only THP-1 cells.

Results: The overall *in vitro* toxicity profiles of ENM were as follows: ZnO was cytotoxic to all cell types at ≥ 50 μg/mL, but did not induce IL-1β. TiO_2_ was not cytotoxic except for the nanobelt form, which was cytotoxic and induced significant IL-1β production in THP-1 cells. MWCNTs did not produce cytotoxicity, but stimulated lower levels of IL-1β production in THP-1 cells, with the original MWCNT producing the most IL-1β.

Conclusions: The results provide justification for the inclusion of mechanism-linked bioactivity assays along with traditional cytotoxicity assays for *in vitro* screening. In addition, the results suggest that conducting studies with multiple relevant cell types to avoid false-negative outcomes is critical for accurate evaluation of ENM bioactivity.

The worldwide development of innovative products increasingly includes incorporating engineered nanomaterials (ENMs). It is likely that ENM use will become ever more commonplace in the future and provide many benefits, although harm from inadvertent human exposures may also increase. Some challenges in evaluation of ENM safety need to be addressed, especially considering that the pace of ENM development is exceeding the industry’s ability to sufficiently conduct animal safety testing (Maynard et al. 2006; Nel et al. 2006; Oberdörster et al. 2005). To address this issue, one of the goals in the field of nanotoxicology (or nanosafety) is development of *in vitro* assays that are highly predictive of *in vivo* outcomes in order to triage those ENMs that should proceed to *in vivo* testing (Meng et al. 2009; Oberdörster et al. 2005). Unfortunately, the presence of conflicting interlaboratory data on the relative hazard of individual ENMs is a concern for moving the reliability of *in vitro* testing forward. Therefore, the National Institute of Environmental Health Sciences (NIEHS) developed a consortium program of experts in the field of pulmonary toxicology to conduct coordinated *in vitro* and *in vivo* assays with selected well-characterized ENMs. There are a number of potential causes of variability, including sources of ENM, ENM suspension protocols, selection of target cells, end points selected to evaluate ENM bioactivity, and details of cell culture and end point assays.

All consortium program participants (CPPs) received characterized ENMs for use in this study, including metal oxide nanospheres [rutile/anatase titanium dioxide (TiO_2_-P25), TiO_2_ anatase (TiO_2_-A), and zinc oxide (ZnO)] and high-aspect-ratio materials [multiwalled carbon nanotubes (MWCNTs) and TiO_2_ nanobelts (TiO_2_-NBs)]. Furthermore, the CPPs tested three forms of MWCNT [original (O-MWCNT), purified (P-MWCNT) and functionalized (carboxylated) (F-MWCNT)]. The consortium chose cell lines for the assays based on respiratory tract exposure, thus, the cell types most likely to interact with ENM after deposition. As such, the cell lines included RLE-6TN (rat type II alveolar epithelial cell line), BEAS-2B (human bronchial epithelial cell line), and THP-1 (human monocyte/macrophage cell line) (Dostert et al. 2008; Driscoll et al. 1995; Xia et al. 2008b). The consortium developed ENM suspension protocols (in cell culture media) that were sufficiently reproducible among laboratories (Ji et al. 2010; Porter et al. 2008; Sager et al. 2007). The cell assays included traditional cytotoxicity testing (Xia et al. 2008b) and evaluation of the Nod-like receptor protein 3 (NLRP3) inflammasome activation with THP-1 cells (Hamilton et al. 2009). The consortium chose the inflammasome assay on the basis of evidence that a number of particles such as crystalline silica, asbestos, uric acid crystals, and cholesterol crystals activate the NLRP3 inflammasome, causing the release of interleukin (IL)-1β and IL-18 that have been linked to lung pathology (Cassel et al. 2008; Dostert et al. 2008; Franchi et al. 2009; Tschopp and Schroder 2010). For example, one of the CPP laboratories recently showed that TiO_2_-NBs stimulate the NLRP3 inflammasome in primary murine macrophages (Hamilton et al. 2009). Here we describe the outcomes of the consortium’s *in vitro* studies, and Bonner et al. (2013) describe the *in vivo* studies conducted in rodents using the same well-characterized ENMs.

In the present study, the CPPs identified and minimized critical aspects of current ENM testing protocols that will potentially decrease the variability in reported outcomes from the various laboratories engaged in the field. In addition, the results of this study provide new information on the relative *in vitro* bioactivity of a large group of diverse ENMs that can be used to inform future strategies for *in vitro* testing and for predicting *in vivo* outcomes.

## Materials and Methods

*ENMs and reagents*. The CPPs obtained ZnO from Meliorum Technologies Inc. (Rochester, NY). TiO_2_-P25 (81% anatase and 19% rutile) was purchased from Evonik (Parsippany, NJ); TiO_2_-A was provided by P. Biswas (Washington University, St. Louis, MO); and the CPPs prepared the TiO_2_-NBs as previously described (Hamilton et al. 2009). The CPPs obtained the O-MWCNT stock in powder form from Cheap Tubes Inc. (Brattleboro, VT); obtained the P-MWCNT by treating O-MWCNT with dilute acids, chelating agents, and mild conditions to minimize oxidized or damaged tubes; and created F-MWCNT through further acid treatment of P-MWCNT, which introduced carboxyl groups on 5.27% of the carbon backbone (on a per weight basis) (Chen and Mitra 2008; Wang et al. 2011).

The CPPs purchased low-endotoxin bovine serum albumin (BSA) from Gemini Bio-Products (West Sacramento, CA); dipalmitoylphosphatidylcholine, phorbol 12-myristate, 13-acetate (PMA), and lipopolysaccharide (LPS from *Escherichia coli* 0127:B8) from Sigma-Aldrich (St. Louis, MO); and 1,25-dihydroxy-vitamin D_3_ from EMD Millipore (Billerica, MA). The CPPs purchased the cytotoxicity assays CellTiter 96 (MTS assay) and CytoTox 96 [LDH (lactate dehydrogenase) assay] from Promega (Madison, WI).

*Preparation of ENMs in cell culture media.* The CPPs prepared ENM stock solutions (5 mg/mL) from dry powder using endotoxin-free sterile water and then prepared all ENM suspensions in cell culture media using the stock solutions as needed. Briefly, the CPPs vortexed and then sonicated ENM stock solutions (with the exception of TiO_2_-NB, which was stirred to prevent mechanical shear) using a water bath sonicator or cup horn sonicator (depending on laboratory availability) immediately before diluting the solutions into complete cell culture media.

*Cell culture and co-incubation with EMN*. The CPPs grew all cells at 37°C in a 5% CO_2_ atmosphere. RLE-6TN cells, a rat alveolar type II epithelial cell line, from American Type Culture Collection (ATCC; Manassas, VA) were cultured in Ham’s F12 medium (ATCC) supplemented with l-glutamine, bovine pituitary extract (BPE), insulin, insulin growth factor (IGF)-1, transferrin, and epithelial growth factor (EGF), supplemented with 10% fetal bovine serum (FBS). THP-1 cells, a human acute monocytic leukemia cell line (ATCC) were cultured in HEPES-buffered RPMI 1640 supplemented with l-glutamine (Mediatech, Corning, NY), 0.05 mM β-mercaptoethanol, and 10% FBS (PAA Laboratories, Dartmouth, MA). BEAS-2B cells (ATCC) were cultured in bronchial epithelial growth medium (BEGM) obtained from Lonza Inc. (Walkersville, MD) supplemented with BPE, insulin, hydrocortisone, human EGF, epinephrine, triiodothyronine, transferrin, gentamicin/amphotericin-B, and retinoic acid. For the THP-1 differentiation performed in the first series of experiments (phase I), the CPPs pretreated cells with 1.62 µM (1 µg/mL) PMA for 18 hr. However, the CPPs identified excessive cell clumping and cell death during the phase I studies. Therefore, the CPPs alternatively pretreated THP-1 cells with vitamin D_3_ at 150 nM overnight and then 5 nM PMA in order to obtain the differentiated macrophage-like cells used during the second series of experiments (phase II). For the IL-1β release, co-culturing THP-1 cells with 10 ng/mL LPS was necessary to initiate transcription of pro-IL-1β. The CPPs initiated aggressive phagocytic activity by adding PMA just before particle exposure.

Before ENM exposure, the CPPs cultured aliquots of 1.5 × 10^4^ cells (for THP-1 cells, 10^5^ cells were seeded into each well of a 96-well plate) in 0.2 mL of the cell culture media in 96-well plates (Costar, Corning, NY) at 37°C for 24 hr. The CPPs freshly prepared all of the ENM suspensions at final concentrations of 10, 25, 50, and 100 µg/mL in the cell culture media. After exposure of the cells to the ENMs for 24 hr at 37°C, the CPPs collected supernatants to measure LDH and IL-1β production then used the remaining cells to test cellular viability by MTS assay.

*Physicochemical characterization of ENMs*. The CPPs identified the primary particle size and morphology of the ENMs by using a transmission electron microscope (TEM; model 100CX) and a scanning electron microscope (SEM; model JSM-7600F) (both from JEOL Ltd., Tokyo, Japan). In addition, the CPPs characterized the particle hydrodynamic size in H_2_O and cell culture media using dynamic light scattering (DLS) (Ji et al. 2010). The CPPs characterized particle crystallinity and structure using X-ray diffraction measurements and measured particle surface area by Brunauer–Emmett–Teller (BET) surface area analysis. The CPPs performed zeta-potential measurements of the ENM suspensions using a ZetaSizer Nano-ZS instrument (Malvern Instruments, Worcestershire WR, UK). Finally, the CPPs determined the elemental composition of the particles as well as ZnO dissolution rate using inductively coupled plasma mass spectrometry (ICP-MS) (model SCIEX Elan DRCII; PerkinElmer, Norwalk, CT).

*Endotoxin analysis of ENMs*. CPPs measured the endotoxin content of ENM stock suspensions, as well as dispersions in PBS and tissue culture media, using the colorimetric Limulus amebocyte lysate assay (Lonza Inc.). The LPS content of all ENM suspensions was < 0.3 EU/mL.

*Determination of cell viability*. The CPPs determined cellular viability using MTS (CellTiter 96) and LDH (CytoTox 96; both from Promega) according to the manufacturer’s protocols. To avoid the interference created by ENMs while measuring formazan absorbance at 490 nm, the CPPs introduced a centrifugation (2000 × *g* for 10 min) procedure in phase II experiments to collect particles in the wells after incubation with the MTS reagents. CPPs then followed this centrifugation step with a brief mixing and transfer of the supernatant to a new 96-well plate before measuring the formazan absorbance at 490 nm. The CPPs eliminated interference of any residual LDH in FBS by heat-inactivation (70°C water bath for 5 min).

*ELISA for IL-1*β *quantification*. The CPPs determined IL-1β production in the THP-1 culture supernatant using a human IL-1β ELISA kit (R&D Systems Human IL-1β DuoSet™; R&D Systems, Minneapolis, MN) following the manufacturer’s instructions.

*Statistical analysis*. The CPPs used the two-way analysis of variance followed by Tukey or Bonferroni correction for multiple comparisons of means for statistical analysis of responses across ENMs and cell lines. In order to define interlaboratory comparisons across two harmonization rounds, the CPPs conducted a meta-analysis of LDH, MTS, and IL-1β assays across eight different laboratories for three cell lines (BEAS-2B, RLE-6TN, and THP-1) exposed to several ENMs (TiO_2_-P25, TiO_2_-A, TiO_2_-NBs, ZnO, O-MWCNT, P-MWCNT, and F-MWCNT). The CPPs combined information within assays and cell lines using a robust two-stage hierarchical model of toxicity. For all quantities of interest, the CPPs obtained Monte Carlo inference by implementing a custom Gibbs sampler in the R computing environment (R Foundation for Statistical Computing, Vienna, Austria). To normalize data, the CPPs subtracted background negative control values (MTS, LDH, and IL-1β) and provided adjustments for positive control values in the case of LDH assays. Details about the statistical model used for analysis are provided in Supplemental Material, p. 8 (http://dx.doi.org/10.1289/ehp.1306561).

## Results

**Figure 1:**
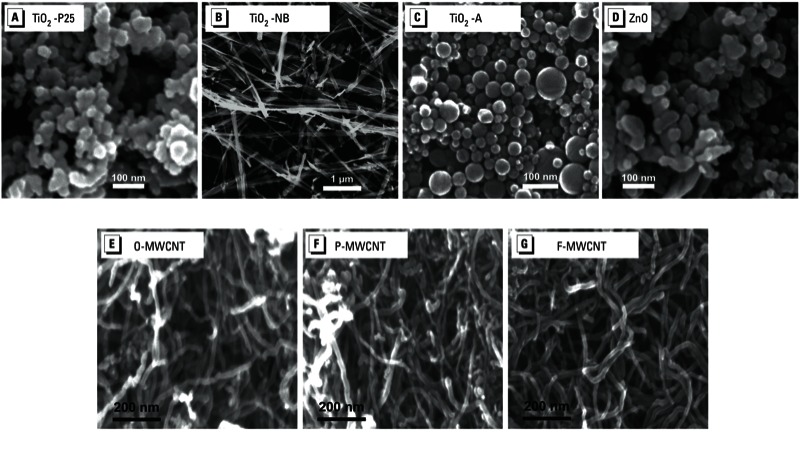
SEM images of ENMs: (*A*) TiO_2_-P25, (*B*) TiO_2_-NB, (*C*) TiO_2_-A, (*D*) ZnO, (*E*) O-MWCNT, (*F*) P-MWCNT, and (*G*) F-MWCNT.

*Physicochemical characterization of consortium ENMs*. The consortium selected two types of nanomaterials for the studies in order to evaluate a sufficient number of material types: *a*) metal oxides (TiO_2_-P25, TiO_2_-A, TiO_2_-NBs, and ZnO), and *b*) three multiwall carbon nanotubes (O-MWCNT, P-MWCNT, and F-MWCNT). A key element of this study was that all of the investigators used the same sets of ENMs, which had been extensively characterized *a priori*. Representative SEM images show the morphology of the different ENMs: TiO_2_-P25, TiO_2_-A, and ZnO were spherical; TiO_2_-NBs were straight, long fibers; O-MWCNT, P-MWCNT, and F-MWCNT were long fibers (Figure 1). The CPPs additionally characterized the ENMs to determine size, surface area, crystal structure, and purity (Tables 1 and 2). To determine the hydrodynamic size and charge of these materials, the CPPs used both water and cell culture media, respectively [in water, Tables 1 and 2; for in media, see Supplemental Material, Tables S1 and S2 (http://dx.doi.org/10.1289/ehp.1306561)]. Functionalization of MWCNT increased the negative surface charge in water, as expected. It should be noted that hydrodynamic diameters for nonspherical particles such as nanotubes and nanowires are defined as the equivalent spherical diameters (i.e., the diameter of a sphere with the same translational diffusion coefficient) and cannot be simply related to the exact particle sizes. Therefore, the hydrodynamic diameters measured here for TiO_2_-NBs and MWCNTs only represent their “relative” sizes. DLS measurements provide a good indication of the dispersion state of high-aspect-ratio ENMs such as MWCNTs (Wang et al. 2010) and cerium oxide (CeO_2_) nanorods and nanowires (Ji et al. 2012). For MWCNTs, the CPPs performed additional experiments using BEGM medium (for BEAS-2B cells), showing that the combination of BSA and 1,2-dipalmitoyl-*sn*-glycero-3 phosphocholine (DPPC) provided optimal suspension stability (see Supplemental Material, Figure S1A). In addition, because ZnO has been shown to dissolve in aqueous solutions, the CPPs determined the dissolution rate of ZnO in water and BEGM and DMEM media. At 24 hr, the percentage of dissolution was 12%, 32%, and 35%, respectively (see Supplemental Material, Figure S1B). Detailed protocols and extra supplementary raw data are available online [Center for Environmental Health Sciences (CEHS) 2012].

**Table 1:** Physicochemical characterization of TiO2 and ZnO ENMs.

Quality	Technique	TiO_2_-P25	TiO_2_-A	TiO_2_-NBs	ZnO
Size (nm)	TEM	~ 24	~ 28	L:7000; W:200; T:10	~ 20
Size in H_2_O (intensity-based) (nm ± SD)	DLS	209 ± 8 (PdI 0.065)	292 ± 70	2,897 ± 117	215 ± 15 (PdI 0.033)
Phase and structure	XRD	81% anatase and 19% rutile	100% anatase	100% anatase	100% zincite
Shape/morphology	TEM	Spheroid	Spherical	Belt	Spheroid
Surface area (m^2^/g)	BET	53	173	18	26
Zeta potential in H_2_O at pH 6.0 (mV ± SD)	Zetasizer	–34.4 ± 1.6	–30.7 ± 0.8	–30.3 ± 2.8	–28.2 ± 0.5
Elemental analysis (weight percent)	ICP-MS	98.6	NA	NA	99.3
Abbreviations: L, length; NA, not available; PdI, polydispersity index; T, thickness; W, width; XRD, X-ray defraction.					

**Table 2:** Physicochemical characterization of MWCNTs.

Quality	Technique	O-MWCNT	P-MWCNT	F-MWCNT
Size (nm)	TEM	D: 20–30; L: 5,000–10,000	D: 20–30; L: 5,000–10,000	D: 20–30; L: 5,000–10,000
Size in H_2_O (intensity-based) (nm ± SD)	DLS	324 ± 33	858 ± 58	234 ± 24
Shape/morphology	TEM	Nanotube	Nanotube	Nanotube
Surface area (m^2^/g)	BET	180	513	26
Zeta potential in H_2_O at pH 6.0 (mV ± SD)	Zetasizer	–12.1 ± 0.3	–11.8 ± 1.1	–48.4 ± 1.7
Elemental analysis (weight percent)	ICP-MS	4.5% Ni, 0.8% Fe	1.8% Ni, 0.1% Fe	0.2% S
Abbreviations: D, diameter; Fe, iron; L, length; Ni, nickel, S, sulfur.				

*Cytotoxicity of ENMs*. *In vitro* studies with ENMs involved eight laboratories and included two phases. In some cases not all eight laboratories were able to report full results. For instance, some optical density readings were > 2.0, and some laboratories had 100% cell lysis values lower than the particle-exposed values. These data were not usable and were not included in the final results. The CPPs carried out phase I studies with previously established protocols used in the respective laboratories of the consortium members. In contrast, the CPPs conducted phase II studies using protocols developed after identifying and solving technical problems in phase I. The CPPs selected MTS and LDH assays because they are the most commonly used single end point cell viability assays for nanotoxicity studies in the literature (Smith et al. 2011; Wang et al. 2010; Xia et al. 2008a). The relative simplicity of the assay procedures allowed the studies to be conducted concurrently.

**Figure 2:**
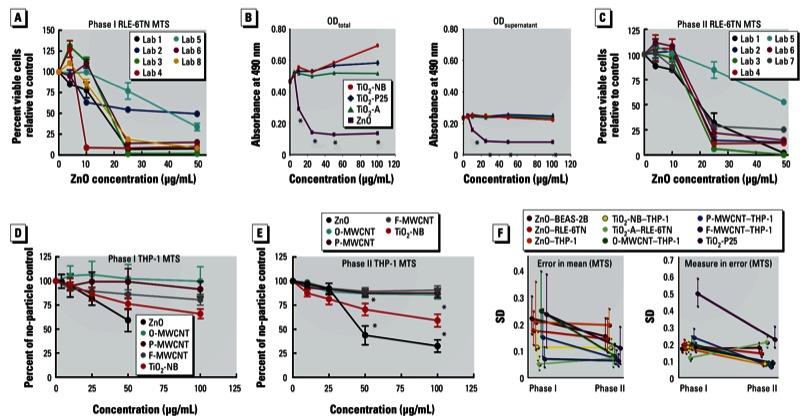
Phase I/II comparisons for RLE-6TN and THP-1 cells using MTS assay data. (*A*) Percent viable RLE-6TN cells relative to no-particle control for each individual laboratory in phase I. (*B*) The ENM distorted OD readings in the MTS assay: with the ENM in the culture well (left); with the media supernatant removed and replaced in wells without particle interference (right); OD, optical density. (*C*) Percent viable RLE-6TN cells relative to no-particle control for each individual laboratory in phase II. (*D,E*) Percent viable cells relative to no-particle control for THP-1 phase I conditions (*D*) and for THP-1 phase II conditions (*E*). (*F*) Changes in error of the mean (left) and measure in error (right) from phase I to phase II trials for MTS assay data. Data are expressed as mean ± SE. *p < 0.05 compared with other particles at the same concentration and/or the “no-particle” control.

*MTS assays*. The CPPs discovered substantial variations among the replicates within individual laboratories in the phase I MTS assays using BEAS-2B and RLE-6TN cells. Figure 2A shows the low consistency for ZnO MTS data using RLE-6TN cells in phase I experiments. The CPPs determined that ENMs interfered directly with the optical density readings (Figure 2B, left). Therefore, the CPPs eliminated the optical interference by adding a centrifugation procedure to isolate the suspended ENM at the bottom of the cell culture dish, then transferring the supernatants to a fresh plate to conduct the absorbance readings (Figure 2B, right). Figure 2C shows the phase II results of MTS assay in RLE-6TN cells using the updated protocols, and the results demonstrated high consistency with low variability among different laboratories.

Figure 2D,E shows the combined data for the MTS assays using THP-1 cells. When comparing the ENMs within each group, the CPPs determined that ZnO was the most toxic followed by TiO_2_-NB. None of the other ENMs caused any significant toxicity as detected by the MTS assay. The combined MTS data for the BEAS-2B and RLE-6TN cells from all laboratories also showed clear toxicity trends for ZnO [see Supplemental Material, Figures S2A,C and S3A,C (http://dx.doi.org/10.1289/ehp.1306561)].

Figure 2F compares reproducibility of MTS assays between phases I and II. Stratifying by ENM and cell line, the CPPs found that both error in mean and measurement error were either reduced or left unchanged when comparing phase I with phase II data. Overall, estimations show a 30% (95% CI: 5, 51%) reduction in the error in mean between phase I and phase II, with significant improvement in reproducibility among laboratories. In addition, a 40% (95% CI: 13, 60%) reduction in measurement error occurred between phase I and phase II, showing substantially improved within-laboratory repeatability.

**Figure 3:**
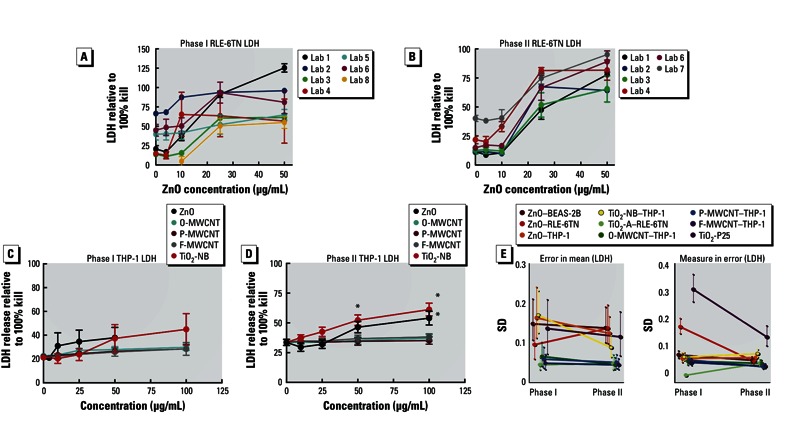
Phase I/II comparisons for RLE-6TN and THP-1 cells using LDH assay data. (*A,B*) Percent LDH release in RLE-6TN cells relative to total cell lysis (100% cell death) for each individual laboratory in phase I (*A*) and in phase II (*B*). (*C,D*) Percent LDH release relative to total lysis for THP-1 phase I conditions (*C*) and for THP-1 phase II conditions (*D*). (*E*) Changes in error of the mean (left) and measure in error (right) from phase I to phase II trials for LDH assay data. Data are expressed as mean ± SE. *p < 0.05 compared with other particles at the same concentration and/or the “no-particle” control.

*LDH assays*. Similar to the MTS assays, the LDH assay results in response to ZnO treatment of RLE-6TN cells improved in the second phase of testing. Three laboratories showed high baseline levels in LDH in phase I as compared with other laboratories and did not have the same dose–response patterns exhibited by the majority of the laboratories (Figure 3A). In contrast, the procedural improvements in phase II show a much better agreement between laboratories (Figure 3B). The combined data for the LDH assays using THP-1 cells showed the same patterns of toxicity compared with the MTS assays (Figure 3C,D). The only two ENMs that achieved significant toxicity using the LDH assay were TiO_2_-NB and ZnO. Detailed summary LDH data for BEAS-2B and RLE-6TN cells can be found in Supplemental Material, Figures S2B,D and S3B,D (http://dx.doi.org/10.1289/ehp.1306561).

Figure 3E reports the results comparing the reproducibility of LDH assays between phases I and II. Stratifying by ENM and cell line, the CPPs found that both error in mean and measurement error were either reduced or left unchanged when comparing phase II with phase I data. Only one ENM by cell line combination (ZnO–RLE-6TN) exhibited decreased reproducibility on average, but the difference in errors was not statistically significant. Comparatively, for the same particle by cell line combination, estimations of measurement error showed a substantial reduction (40% decrease). Overall, assessments of the improvement in assay performance showed a 13% (95% CI: –0.12, 42%) improvement in reproducibility and 21% (95% CI: –0.09, 53%) improvement in repeatability.

**Figure 4:**
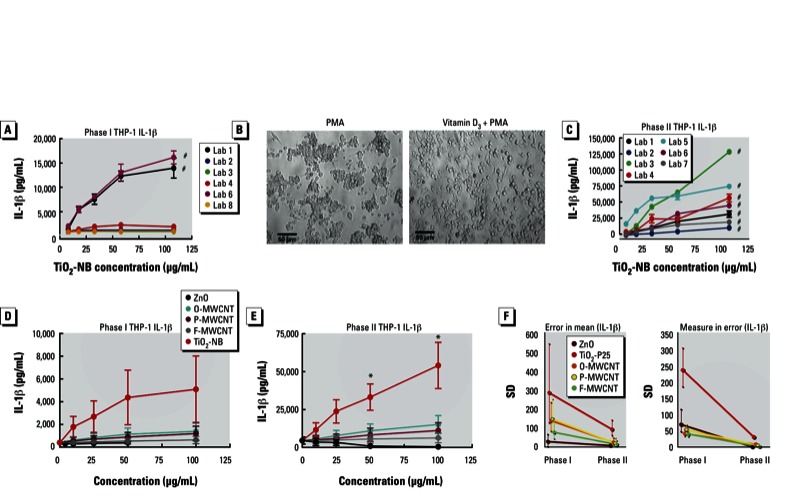
Phase I/II comparisons for THP-1 cells using IL-1β assay data. (*A*) Percent IL-1β release from THP-1 cells for each individual laboratory in phase I. (*B*) THP-1 cell differentiation technique altered cell morphology: THP-1 cells pretreated with 1.62 µM PMA for 24 hr formed clumps (left), whereas cells pretreated with vitamin D3 overnight and then treated with 5 nM PMA were evenly dispersed (right). (*C*) IL-1β release from THP-1 cells for each individual laboratory in phase II. (*D,E*) Summary IL-1β release for phase I conditions (*D*) and for phase II conditions (*E*). (*F*) Changes in error of the mean (left) and measure in error (right) from phase I to phase II trials for IL-1β assay data. Data are expressed as mean ± SE. *p < 0.05 compared with other particles at the same concentration and/or the “no-particle” control. ^#^*p* < 0.001 for dose response (each laboratory’s data analyzed independently).

*Inflammatory potential of ENMs*. The above studies demonstrated that only ZnO (all models tested) and TiO_2_-NB (THP-1 only) caused *in vitro* cytotoxicity. Because cytotoxicity does not necessarily translate mechanistically to *in vivo* pathology, the consortium studies included measurement of NLRP3 inflammasome generated IL-1β from THP-1 cells. The results of the phase I experiments measuring IL-1β production using THP-1 cells demonstrated that only two laboratories showed a significant increase from TiO_2_-NB treatment (Figure 4A). The CPPs determined that the interlaboratory inconsistency in the phase I experimental results was possibly due to cell clumping and excessive cell death when using PMA alone for differentiation (Figure 4B, left). In order to improve data consistency, the CPPs modified the macrophage differentiation method by using vitamin D_3_ followed by a lower concentration of PMA (Figure 4B, right). Figure 4C shows that after using the improved protocols in phase II studies, all laboratories reported significant increases in IL-1β production in THP-1 cells in response to TiO_2_-NB.

Figure 4D,E shows the combined data from the phase I and phase II studies, respectively. The large error bars in phase I were due to the sizeable between-laboratory variation shown in Figure 4A. Consequently, only TiO_2_-NB caused a nonsignificant dose-dependent IL-1β release. In contrast, in phase II the combined data demonstrated that TiO_2_-NB caused a significant IL-1β release from THP-1 cells and MWCNTs also increased IL-1β release, although the results did not achieve significance when all laboratories were averaged. The between-laboratory variance and the large effect of the TiO_2_-NBs negated any significance of the MWCNT IL-1β dose-dependent increases shown in Figure 4E. However, consideration of the individual laboratory data showed a significant dose-dependent increase for the O-MWCNT in all but two laboratories [see Supplemental Material, Figure S4 (http://dx.doi.org/10.1289/ehp.1306561)]. In every case, ranking the bioactivity of the MWCNTs resulted in the following order: O-MWCNT > P-MWCNT > F-MWCNT. The probability of this rank order occurring in every laboratory by chance was *p* < 0.001. In contrast ZnO, which was the most cytotoxic of the ENMs, did not cause any apparent IL-1β release regardless of dose (Figure 4E).

Figure 4F shows the results comparing reproducibility of IL-1β assays between phases I and II. Stratifying by ENM, comparisons of phase I and phase II data show a reduction in both error in mean and measurement error. Combination of all ENMs and cell lines showed an estimated 74% (95% CI: 41, 95%) reduction in the error in mean and significant improvement in reproducibility among laboratories. Furthermore, within-laboratory repeatability improved substantially with an 83% (95% CI: 63, 99%) reduction in measurement error.

## Discussion

The NIEHS consortium conducted this study in an effort to identify and minimize sources of variability among laboratories performing *in vitro* testing of ENMs. Eight laboratories participated in all or part of two rounds (phase I and phase II) of *in vitro* tests using cell lines and ENM types that were collectively selected by consortium members. After identification of several technical problems in phase I, the CPPs significantly improved the interlaboratory variability in phase II. Furthermore, conclusions on the relative potency of the different ENMs for both toxicity and bioactivity will contribute to the information necessary to help predict relative risk for the ENMs. In addition, results showed the MTS assay to be comparable in data quality and predictive value when compared with the LDH assay for toxicity testing.

A significant finding of this study was that the development of harmonized *in vitro* assay protocols made it possible to achieve reproducible results among different laboratories. This study was among the first attempts of large scale round-robin tests of ENMs at the national and international level. There have been reports of a successful effort to perform interlaboratory comparisons to characterize ENMs by establishing detailed shipping, measurement, and reporting protocols (Lamberty et al. 2011; Roebben et al. 2011). Specifically, researchers demonstrated a reduction in interlaboratory variations, especially for dynamic scattering measurements (Roebben et al. 2011). In comparison, the present study outlined a novel method of *in vitro* testing using multiple cell types, ENMs, and assays.

The planning of these *in vitro* tests included steps to control for many factors. For example, protocol development included consideration of varying cell culture conditions, cell type, cell number, and cell viability, assay protocols, and most important, experience of the scientist that actually performed the experiments. To this end, the CPPs obtained cells and reagents from the same batch or lot number and followed a detailed cell culture and assay protocol for each experiment. Investigators and laboratory personnel established a mechanism to communicate with others and to share their experience in performing specific assays. The frequent communication was very helpful for achieving reproducible results within and among the laboratories.

The strategies established for interlaboratory communication provided a foundation for developing more effective protocols during phase II studies to account for the inherent difficulties in evaluating ENMs. For example, through close communication, the CPPs determined that ENMs interfere with optical density readings—creating artifacts and erroneous high MTS values (Kroll et al. 2012). The phase I MTS assay protocol, as based on the Promega manufacturer’s instructions, did not take into consideration potential absorbance anomalies created by the ENMs. As a result, although the combined phase I MTS assays showed clear toxicity profiles of TiO_2_-P25, TiO_2_-A, TiO_2_-NB, and ZnO in RLE-6TN and BEAS-2B cells [see Supplemental Material, Figures S2 and S3 (http://dx.doi.org/10.1289/ehp.1306561)], a large variation existed among the laboratories, as shown in Figure 2A. The CPPs determined this anomaly to be due to the presence of residual ENM following treatment of the cells, as demonstrated in Figure 2B. Therefore, the phase II protocol introduced a simple centrifugation step and media removal after MTS reagent incubation, thus allowing the absorbance to be read using supernatant containing ENM-free MTS solution. This additional step eliminated the ENM interference with the assay and significantly improved the intra- and interlaboratory consistency.

The CPPs determined ENM interference to be only one potential source of variability for *in vitro* assays. Phase I results of the LDH and IL-1β assays clearly demonstrated that cell culture conditions also contributed to the observed variability. Stimulated release of IL-1β from THP-1 cells relied in part on the activation and differentiation of THP-1 cells by high levels of PMA in the phase I protocol. PMA induced strong responses in THP-1 cells, resulting in severe clumping of the THP-1 cells during the activation process, in addition to excessive cell death occurring upon scraping the cells. Using this protocol, only two laboratories demonstrated toxicity of TiO_2_-NBs in the MTS and LDH assays or inflammasome activation in the IL-1β assay in the phase I tests. However, by replacing some of the PMA with vitamin D_3_, a milder signal for THP-1 activation, the cells remained well dispersed. Consequently, phase II results demonstrated similar results for IL-1β production by THP-1 cells stimulated with TiO_2_-NBs between laboratories.

Consortium efforts found that the MTS assay was at least equal in reliability to assess ENM toxicity as the LDH assay. Both toxicity assays tracked each other very well for the different ENMs and cell types. Although this finding needs further validation, it suggests that the easy-to-conduct MTS assay could serve as a highly reliable testing method for *in vitro* studies of ENM-induced cytotoxicity when conducted as described in the phase II protocol. Supplemental information is available online (CEHS 2012). The observation of a difference in the relative potency between TiO_2_-NB and ZnO between the two assays is likely not as important as the observation that both were significantly cytotoxic *in vitro*, suggesting that both ENMs should be tested *in vivo*.

The consortium studies also provided reproducible findings of relative toxicity and bioactivity of the various ENMs across the different cell lines. For example, ZnO was highly toxic to all three cell types examined in this study, yet it did not stimulate inflammasome activity in THP-1 cells (Figure 4), suggesting that cytotoxicity and bioactivity (e.g., stimulation of inflammasome-mediated IL-1β release) do not necessarily correlate. Studies on inflammation and lung pathology implicate the importance of inflammasome-generated IL-1β production (Cassel et al. 2008; Dostert et al. 2008; Franchi et al. 2009; Tschopp and Schroder 2010). In addition, a consortium laboratory recently demonstrated the importance of IL-1β release in generating an inflammatory response to MWCNTs *in vivo* (Girtsman et al. 2012). Therefore, the consortium recommends the inclusion of mechanism-linked bioactivity assays along with traditional cytotoxicity assays for *in vitro* ENM screening.

The two forms of TiO_2_ nanospheres (P25 and A) were not significantly cytotoxic or bioactive, yet TiO_2_-NBs were highly cytotoxic and selectively induced inflammasome activity toward THP-1 cells, consistent with previous studies (Hamilton et al. 2009). The lack of toxicity of the TiO_2_-NBs toward epithelial cells can probably be explained by their poor internalization of the material, and suggests that if *in vitro* studies were conducted solely with epithelial cell lines such as those used in this study, the high toxicity of this material would not have been identified. Consequently, an important recommendation is to conduct studies with multiple relevant cell types to avoid false-negative outcomes.

In parallel with the *in vitro* studies, another consortium effort among seven different laboratories conducted *in vivo* studies using rats, mice, and most of the ENMs described in the present study. These researchers performed similar efforts to address technical issues and methods to reduce variability (Bonner et al. 2013). The CPPs *in vitro* TiO_2_ studies predicted the *in vivo* outcomes with more laboratories observing significant lung inflammation with the TiO_2_-NBs compared with the two nanosphere forms. The results presented in this study are also consistent with a recent report demonstrating that TiO_2_-NBs, but not nanospheres, caused lung fibrosis (Porter et al. 2013). Furthermore, observations showed a greater activity of O-MWCNT compared with P-MWCNT or F-MWCNT. Therefore, the *in vitro* studies had the potential to accurately predict the relative potency for the TiO_2_ materials observed *in vivo*. In fact, the IL-1β (proxy measure for NLRP3 inflammasome) result from this study would predict that the TiO_2_-NBs and the O-MWCNT would be the two ENMs that would result in the greatest inflammation, and that was confirmed in the corresponding *in vivo* work (Bonner et al. 2013).

## Conclusions

These studies strove to improve concordance of test results among laboratories while ensuring that the assays were predictive of *in vivo* outcome. The CPPs achieved the goal of producing reliable and repeatable *in vitro* toxicity testing for ENMs; however, improving the predictability of the assays is still a work in progress. Care needs to be taken to understand the limitation of *in vitro* testing and not to overinterpret *in vitro* studies without comprehensive companion *in vivo* studies. These studies used well-characterized nanomaterials, including a positive and negative control, in addition to a well-established dispersion protocol ensuring stable suspensions in cell culture media. This consortium effort provided a series of harmonized protocols and tested models for the nanotoxicological field to use. The results also demonstrated that toxicity and inflammasome activity did not always track each other and that different cell types yielded different estimates of safety of different ENMs. Consequently, future studies should utilize multiple end points and multiple cell types to avoid false-negative results. Finally, this effort serves as a good template for future endeavors in the field of nanotoxicology, providing key elements necessary for collaborative efforts between laboratories.

## Supplemental Material

(1.5 MB) PDF
