# Supplementary material for: Interlaboratory Evaluation of in Vitro Cytotoxicity and Inflammatory Responses to Engineered Nanomaterials: The NIEHS Nano GO Consortium
Source: Environ Health Perspect. 2013 May 6;121(6):683–90. doi: 10.1289/ehp.1306561 (PMC3672931; doi:10.1289/ehp.1306561)
Supplement: (1.5 MB) PDF [file ehp.1306561.s001.pdf]

Supplemental Material

Interlaboratory Evaluation of *in Vitro* Cytotoxicity and Inflammatory Responses to Engineered Nanomaterials: The NIEHS NanoGo Consortium

Tian Xia[1], Raymond F. Hamilton Jr[2], James C. Bonner[3], Edward D. Crandall[4], Alison Elder[5], Farnoosh Fazlollahi[4], Teri A. Girtsman[2], Kwang Kim[4], Somenath Mitra[6], Susana A. Ntim[6], Galya Orr[7], Mani Tagmount[8], Alexia J. Taylor[3], Donatello Telesca[1], Ana Tolic[7], Christopher D. Vulpe[8], Andrea J. Walker[5], Xiang Wang[1], Frank A. Witzmann[9], Nianqiang Wu[10], Yumei Xie[7], Jeffery I. Zink[1], Andre Nel[1], and Andrij Holian[2]

[1]Department of Medicine, Division of NanoMedicine, Center for Environmental Implications of Nanotechnology, California Nanosystems Institute, University of California at Los Angeles, Los Angeles, California, USA

[2]Center for Environmental Health Sciences, Department Biomedical and Pharmaceutical Sciences, University of Montana, Missoula, Montana, USA

[3]Department of Environmental and Molecular Toxicology, North Carolina State University, Raleigh, North Carolina, USA

4Department of Medicine, University of Southern California, Los Angeles, California, USA

[5]Department of Environmental Medicine, University of Rochester, Rochester, New York, USA

[6]Department of Chemistry and Environmental Science, New Jersey Institute of Technology, Newark, New Jersey, USA

[7]Environmental Molecular Sciences Laboratory, Pacific Northwest National Laboratory, Richland Washington, USA

[8]Department of Nutritional Science and Toxicology, University of California, Berkeley, Berkeley, California, USA

[9]Department of Cellular & Integrative Physiology, Indiana University School of Medicine, Indianapolis, Indiana, USA
[10]Department of Mechanical and Aerospace Engineering, West Virginia University, Morgantown, West Virginia, USA
Correspondence: Andrij Holian, andrij.holian@mso.umt.edu, 280 Skaggs Building, Center for Environmental Health Sciences, 32 Campus Drive, The University of Montana, Missoula, MT 59812 (406) 243-4018

Table of Contents:

Page 3: Supplemental Material, Table S1: Size and zeta potential of TiO2 and ZnO nanoparticles in media

Page 3: Supplemental Material, Table S2: Size and zeta potential of MWCNT in media

Page 4: Supplemental Material, Figure S1: Stability of O-MWCNT, P-MWCNT, and F-MWCNT suspensions in BEGM in the absence or presence of dispersing agents.

Page 5: Supplemental Material, Figure S2: Cytotoxicity in the BEAS-2B Model.

Page 6: Supplemental Material, Figure S3: Cytotoxicity in the RLE-6TN model.

Page 7: Supplemental Material, Figure S4: Individual lab results and summary results for IL-1β release in the THP-1 model exposed to MWCNT variants.

Page 8: Supplemental Material, Hierarchical Model for Reproducibility Analysis

Supplemental Material, Table S1: Size and zeta potential of TiO2 and ZnO nanoparticles in tissue culture media (mean ±s.d.)

Quality	Technique	P25	Anatase	Nanobelts	ZnO
**Size in BEGM (nm) (intensity-based)**	DLS	374±38	385±85	1765±265	196±13
**Size in F12 (nm) (intensity-based)**	DLS	247±7	200±15	1463±39	371±27
**Size in RPMI (nm) (intensity-based)**	DLS	204±7	546±11	1590±126	227±9
**Zeta Potential in BEGM (mV)**	Zetasizer	-13.6±1.6	-10.9±1.8	-6.7±2.1	-11.0±1.4
**Zeta Potential in F12 (mV)**	Zetasizer	-7.7±2.2	-7.9±2.8	-21.5±1.8	-10.8±3.6
**Zeta Potential in RPMI (mV)**	Zetasizer	-12.8±0.1	-11.3±0.7	-12.7±4.7	-13.5±0.2

Supplemental Material, Table S2: Size and zeta potential of the MWCNT in tissue culture media (mean ±s.d.)

Quality	Technique	O-MWCNT	P-MWCNT	F-MWCNT
**Size in BEGM (nm) (intensity-based)**	DLS	187±51	247±48	163±13
**Size in RPMI (nm) (intensity-based)**	DLS	419±48	375±23	244±4
**Zeta Potential in BEGM (mV)**	Zetasizer	-11.8±1.4	-10.5±1.1	-9.9±1.6
**Zeta Potential in RPMI (mV)**	Zetasizer	-10.5±0.9	-9.8±1.1	-11.4±1.3

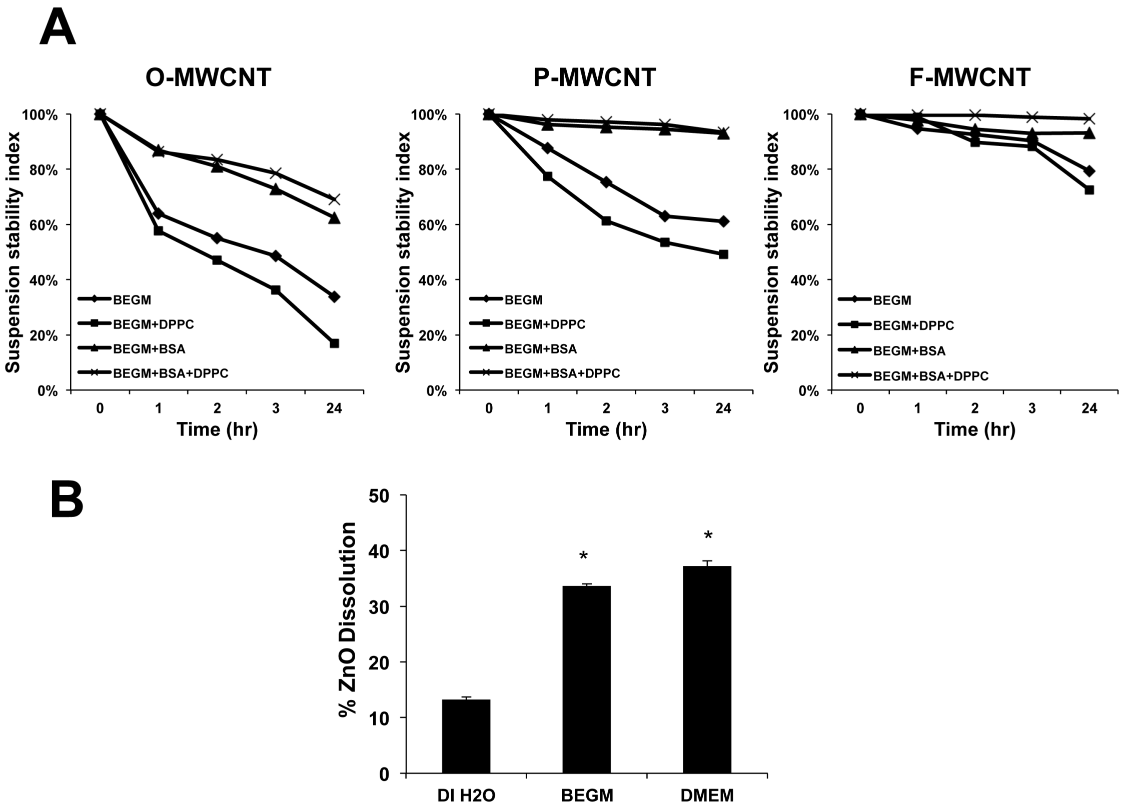

Supplemental Material, Figure S1: Stability of O-MWCNT, P-MWCNT, and F-MWCNT suspensions in BEGM in the absence or presence of dispersing agents [BSA (0.6 mg/mL)±DPPC (0.01 mg/mL)]. (A) The suspension stability index of the MWCNT was determined as a function of time after suspension at 50 μg/mL in BEGM in the absence or presence of BSA, DPPC, or BSA+DPPC. The suspension stability index was calculated as the % of initial MWCNT absorbance (t = 0) at λ=550 nm for time periods of 1, 2, 3, and 24 h. The absorbance measurements were carried out by a UVvis spectrometer (SpectroMax M5e, Molecular Devices Corp., Sunnyvale, CA). (B) The dissolution rate of ZnO in DI H_2O, BEGM, and DMEM media. The ZnO dissolution was determined by ICP-MS: 50 μg/mL nanoparticles was suspended in DI H_2O, BEGM, and DMEM media at room temperature for 24 h. The suspension was centrifuged at 20,000 g for 1 h, and the zinc concentration in the supernatant was determined by ICP-MS. Data are expressed as means ± *SEM*; * indicates significance at $P < 0.05$ compared to the dissolution rate of ZnO in DI H_2O.

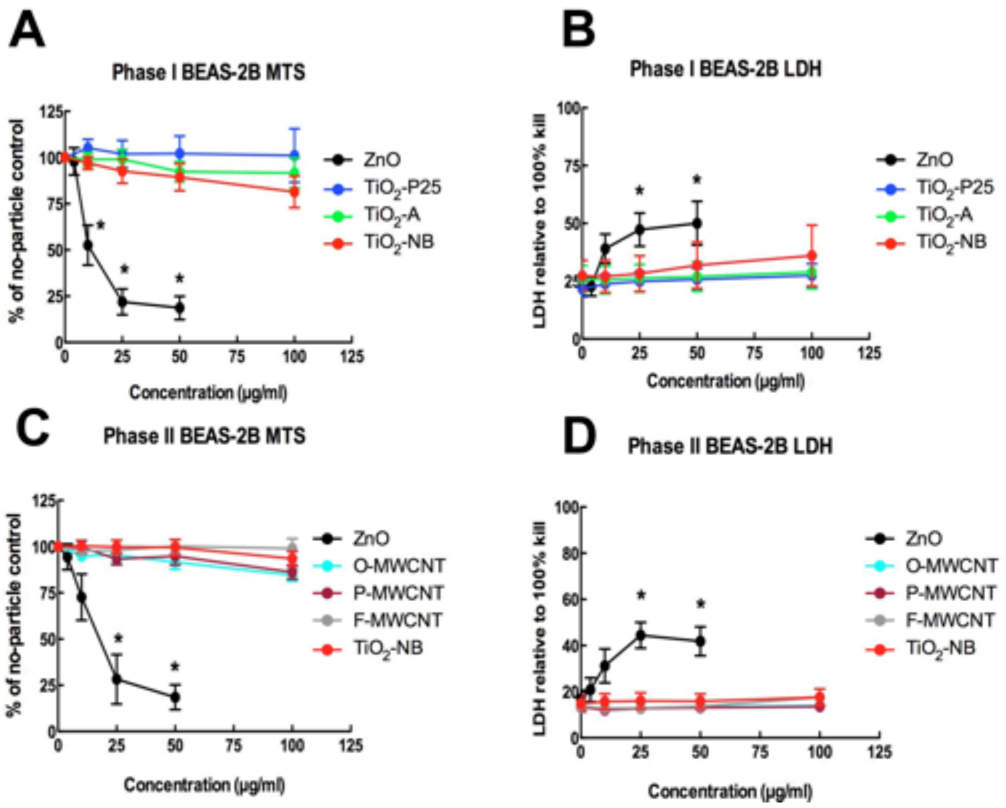

Supplemental Material, Figure S2: Cytotoxicity in the BEAS-2B Model. A) Percent viable cells relative to no particle control for BEAS-2B Phase I conditions. B) Percent LDH release relative to total lysis (100% cell death) for BEAS-2B Phase I conditions. C) Percent viable cells relative to no particle control for BEAS-2B Phase II conditions. D) Percent LDH release relative to total lysis (100% cell death) for BEAS-2B Phase II conditions. Data are expressed as means ± *SEM*; * indicates significance at $P < 0.05$ compared to other particles at the same concentration and the "no particle" control.

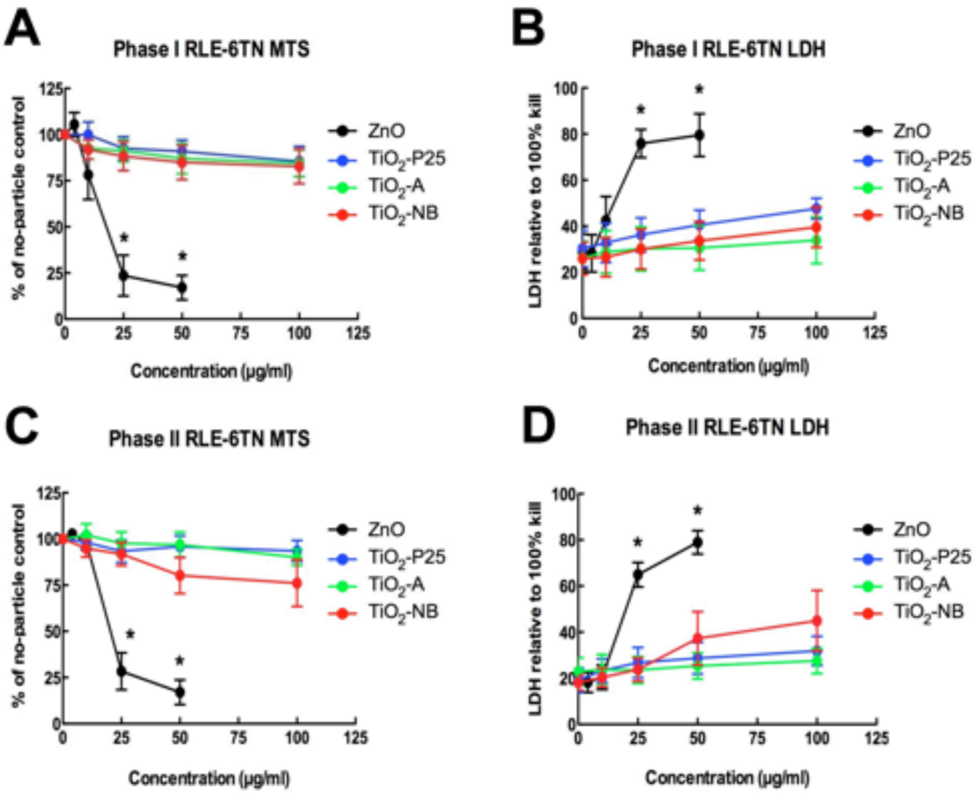

Supplemental Material, Figure S3: Cytotoxicity in the RLE-6TN model. A) Percent viable cells relative to no particle control for RLE-6TN Phase I conditions. B) Percent LDH release relative to total lysis (100% cell death) for RLE-6TN Phase I conditions. C) Percent viable cells relative to no particle control for RLE-6TN Phase II conditions. D) Percent LDH release relative to total lysis (100% cell death) for RLE-6TN Phase II conditions. Data are expressed as means ± *SEM*; * indicates significance at $P < 0.05$ compared to other particles at the same concentration and the “no particle” control.

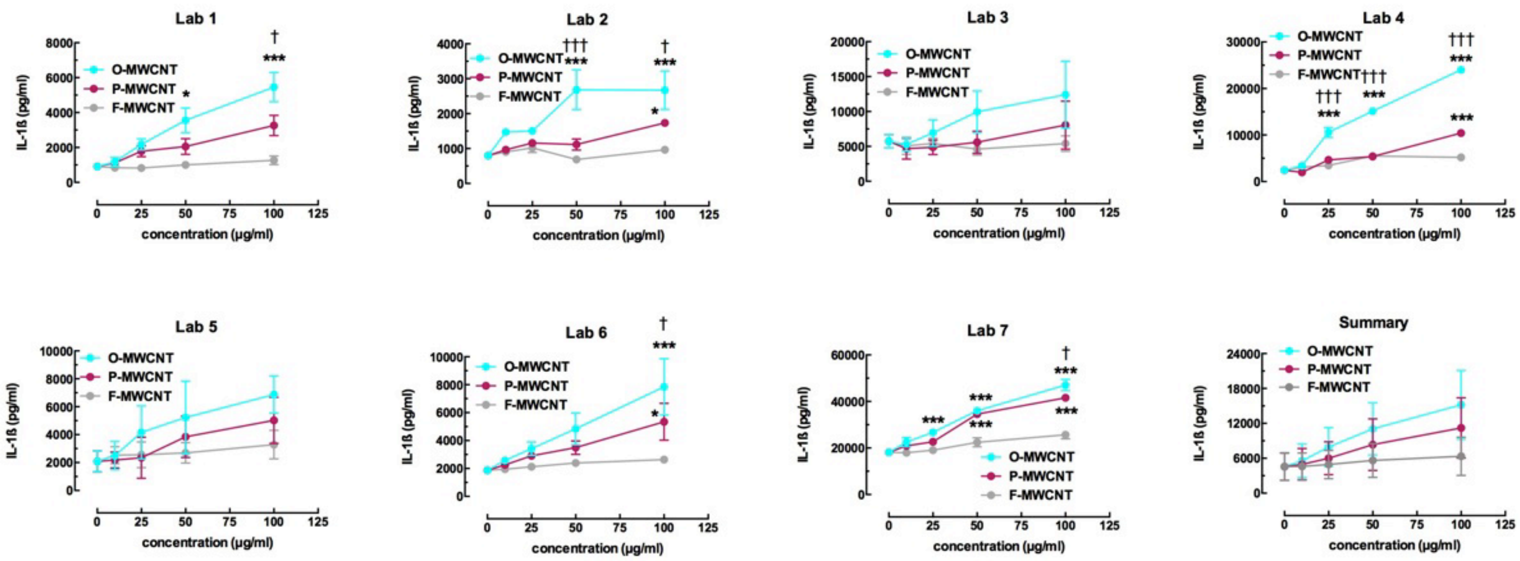

Supplemental Material, Figure S4: Individual lab results and summary results for IL-1β release in the THP-1 model exposed to MWCNT variants. Data are expressed as means ± *SEM*; asterisks indicate significance at *** $P < 0.001$, ** $P < 0.01$, or * $P < 0.05$ compared to F-MWCNT at the same concentration. Daggers indicate significance at ††† $P < 0.001$, †† $P < 0.01$, or † $P < 0.05$ compared to P-MWCNT at the same concentration.

Supplemental Material, Hierarchical Model for Reproducibility Analysis

Within assay, particle and cell line, let y_{rijk} be the normalized response value measured during round r = 1,2; for lab i = 1,…,8; exposure level j = 1,…,5 and replicate k=1,…,3. We consider the following two stage hierarchical model:

1) $y_{rijk} = m_{rij} + \varepsilon_{rijk}$, with $\varepsilon_{rijk} \sim N(0,\sigma_r^2)$; (Sampling model)

2) $m_{rij} = \mu_{rj} + e_{rij}$, with $e_{rij} \sim N(0,\tau_r^2)$. (Mean model)

In the foregoing formulation, m_{rij} is the mean response over replicates obtained during round r, by lab i, for dose j. The measurement error ε_{rijk} is assumed to be Gaussian with mean zero and variance σ_r^2, assumed to be specific to round r. The mean model in (2) assumes a population mean μ_{rj} that is specific to experimental round r and dose j, but aggregates over labs, therefore being interpreted as the overall mean. The error in mean e_{rij} measures deviations of individual lab means m_{rij} from the overall means μ_{rj} and is assumed to be Gaussian with mean zero and variance τ_r^2.

The model is completed with the following conjugate prior distributions:

1. $\mu_{rj} \sim N(0,\upsilon_\mu)$,
2. $\sigma_r^2 \sim IG(a_\sigma, b_\sigma)$,
3. $\tau_r^2 \sim IG(a_\tau, b_\tau)$.

Our inference centers on two main quantities of interest, namely: the posterior distribution of the measurement error σ_r^2, that we interpret as a measure of repeatability in experimental round r; and the posterior distribution of the error in mean τ_r^2, which we interpret as a measure of experimental reproducibility in round r.

These quantities are estimated with arbitrary precision via Markov chain Monte Carlo simulation. In our analysis we considered diffuse prior information setting $\nu_\mu = 10^8$, $a_\sigma = b_\sigma = a_\tau = b_\tau = 0.1$. Our conclusions are not sensitive to alternative default specification of the prior structure.
